# Using mobile phones to improve young people sexual and reproductive health in low and middle-income countries: a systematic review to identify barriers, facilitators, and range of mHealth solutions

**DOI:** 10.1186/s12978-020-01059-7

**Published:** 2021-01-16

**Authors:** Anam Shahil Feroz, Naureen Akber Ali, Adeel Khoja, Armish Asad, Sarah Saleem

**Affiliations:** 1 Department of Community Health Sciences, The Aga Khan University, Stadium Road, PO Box 3500, Karachi, 74800 Pakistan; 2 School of Nursing and Midwifery, The Aga Khan University, Stadium Road, PO Box 3500, Karachi, 74800 Pakistan; 3 Department of Medicine, The Aga Khan University, Stadium Road, PO Box 3500, Karachi, 74800 Pakistan; 4 Department of Cardiology, Endocrinology and Internal Medicine, Northwest Clinic, Dubai, United Arab Emirates; 5 Institute of Health Policy, Management, and Evaluation, University of Toronto, Toronto, ON Canada

**Keywords:** Mobile phones, mHealth, Young people sexual and reproductive health, Low and middle-income countries, Systematic review, Facilitators, Barriers

## Abstract

**Background:**

Globally, reproductive health programs have used mHealth to provide sexual and reproductive health (SRH) education and services to young people, through diverse communication channels. However, few attempts have been made to systematically review the mHealth programs targeted to improve young people SRH in low-and-middle-income countries (LMICs). This review aims to identify a range of different mHealth solutions that can be used for improving young people SRH in LMICs and highlight facilitators and barriers for adopting mHealth interventions designed to target SRH of young people.

**Methods:**

Databases including PubMed, CINAHL Plus, Science Direct, Cochrane Central, and grey literature were searched between January 01, 2005 and March 31, 2020 to identify various types of mHealth interventions that are used to improve SRH services for young people in LMICs. Of 2948 titles screened after duplication, 374 potentially relevant abstracts were obtained. Out of 374 abstracts, 75 abstracts were shortlisted. Full text of 75 studies were reviewed using a pre-defined data extraction sheet. A total of 15 full-text studies were included in the final analysis.

**Results:**

The final 15 studies were categorized into three main mHealth applications including client education and behavior change communication, data collection and reporting, and financial transactions and incentives. The most reported use of mHealth was for client education and behavior change communication [n = 14, 93%] followed by financial transactions and incentives, and data collection and reporting Little evidence exists on other types of mHealth applications described in Labrique et al. framework. Included studies evaluated the impact of mHealth interventions on access to SRH services (n = 9) and SRH outcomes (n = 6). mHealth interventions in included studies addressed barriers of provider prejudice, stigmatization, discrimination, fear of refusal, lack of privacy, and confidentiality. The studies also identified barriers to uptake of mHealth interventions for SRH including decreased technological literacy, inferior network coverage, and lower linguistic competency.

**Conclusion:**

The review provides detailed information about the implementation of mobile phones at different levels of the healthcare system for improving young people SRH outcomes. This systematic review recommends that barriers to uptake mHealth interventions be adequately addressed to increase the potential use of mobile phones for improving access to SRH awareness and services.

**Systematic review registration:**

PROSPERO CRD42018087585 (Feb 5, 2018)

## **Plain English Language Summary**

In LMICs, most young people aged 10–24 years, have very limited, or no access to sexual and reproductive health (SRH) education and services, largely due to lack of awareness, social stigma, policies and procedures inhibiting provision of contraception and abortion services to girls, and judgmental attitudes of healthcare professionals. Thus, young people have special SRH education needs that remain unmet, and to address these specific SRH needs, the use of innovative and novel approaches are required to ensure access to safe, effective, affordable, and acceptable SRH services. Worldwide, diverse mHealth solutions have been used to connect the young population to SRH information and services. Similarly, mHealth technology can be used in LMICs to reach out to the youth population and to engage them to provide acceptable, safe, cost-effective, and accurate SRH services. This systematic review aims to highlight potential barriers and facilitators for the uptake of mHealth interventions for young people SRH in LMICs. The review has provided an understanding of how mHealth solutions targeted to the youth population help address issues of ‘provider prejudice, stigmatization, discrimination, fear of refusal, lack of privacy and confidentiality, cost prohibitions, and transportation challenges’. The review provides insights for the research community and public health professionals in making decisions regarding the use of innovative, engaging and effective mobile phone interventions to improve young people SRH outcomes.

## Background

In most lower-middle-income countries (LMICs), young people (adolescents and youth), aged 10–24 years, have very limited, or no access to sexual and reproductive health (SRH) education and services. This is largely due to lack of awareness, social stigma, policies, and procedures inhibiting the provision of contraception and abortion services to girls, and judgmental attitudes of healthcare professionals [1, 2]. Thus, young people have special SRH education needs that remain unmet, and to address these specific SRH needs, the use of innovative and novel approaches are required to ensure access to safe, effective, affordable, and acceptable SRH services [2].

mHealth involves the use of mobile technologies and multimedia tools to accomplish health goals and support healthcare delivery [3]. Many LMICs have attained a substantial level of cell phone penetration (over 90%) in recent years [4, 5]. On account of the rapid expansion of cell phone ownership and mobile phone penetration in LMICs, the novel field of mHealth has gained much progress and it is being used rapidly in hundreds of diverse health-related projects [3]. The high mobile phone penetration has led to increase usage of mobile phones, especially amongst younger population in LMICs [6, 7]. Young people are responsive and enthusiastic to use new innovative technologies such as mHealth to address barriers to receiving SRH information and services [8–10]. The mHealth technology can help overcome most of the barriers including provider prejudice, stigmatization, discrimination, fear of refusal, lack of privacy and confidentiality, an embarrassment in seeking SRH education and services on highly sensitive topics, cost prohibitions, and transportation challenges, by providing safe, accurate, cost-effective, timely and tailored SRH services to young people [11]. More importantly, mHealth offers privacy, convenience and easy access in contrast to face-face consultations with healthcare professionals, which eventually addresses the barriers of stigmatization and embarrassment in receiving tailored SRH services [12]. Worldwide, diverse mHealth solutions have been used to connect the young population to SRH information and services [13]. Similarly, mHealth technology can be used in LMICs to reach out to youth population and to engage them to provide acceptable, safe, cost-effective and accurate SRH services [11, 14].

In an effort to tap into the potential of mHealth for young people SRH services, there has been an increase in the amount of research in high-income countries (HIC) in recent years. However, little evidence exists on the use of mHealth interventions for improving young SRH among young people in LMICs. In previous studies, attempts have been made to review the mHealth programs for young people SRH using mHealthevidence.org website and through a global call for collecting information on mHealth interventions [15, 16]. A systematic review by L'Engle and colleagues assessed strategies on using mHealth to improve young people SRH by using the mHealth Evidence Reporting and Assessment (mERA) checklist; although only three out of the 35 articles included in the review were related to LMICs, the small number of articles reflected the lack of literature from LMICs [15]. Another review by Ippoliti & L'Engle summarized 17 projects which involved mHealth interventions to improve young people SRH in LMICs, through the aforementioned global call for information. Both of these reviews included evidence regarding the use of mHealth for improving young people SRH. However, very little is known regarding the potential barriers and facilitators for the uptake of mobile phone interventions for improving young people SRH. This systematic review aims to highlight potential barriers and facilitators for the uptake of mHealth interventions for young people SRH, in LMICs.

Labrique and colleagues identified 12 mHealth applications to respond to various health issues [17]. Few healthcare programs involve one application while others may include two or more mHealth applications for addressing a particular health issue. The classification of 12 mHealth applications as per Labrique and colleagues is illustrated in Table 1. A similar framework is being used to categorize the range of mHealth interventions that can be used to improve young people SRH.

**Table 1:** Twelve common mHealth applications

1. Client education and behavior change communication (BCC)	
2. Sensors and point-of-care diagnostics	
3. Registries/vital events tracking	
4. Data collection and reporting	
5. Electronic health records	
6. Electronic decision support (information, protocols, algorithms, checklists)	
7. Provider to provider communication (user groups and consultation)	
8. Provider work planning and scheduling	
9. Provider training and education	
10. Human Resource management	
11. Supply chain management	
12. Financial transactions and incentives	

## Methods

The objectives of the review are twofold:

1. To report the range of mHealth solutions which can be used for improving young people SRH
2. To report facilitating and impeding factors for the uptake of mHealth interventions for young people SRH

### Eligibility criteria

#### Participants

Studies involving young people (adolescents and youth) aged 10–24 years to which mHealth interventions were delivered for improving their SRH outcomes were included in this review.

#### Settings

LMICs were selected according to the World Bank’s (WB) 2018 Country Classification lists [18]. According to WB, LMICs are those with a Gross National Income (GNI) per capita between USD 996 and USD 3895. Issues concerning the use of mobile phones for young people SRH are common across many LMICs [16]; thus these studies are more comparable than those representing HIC.

#### Intervention and outcomes

Those studies were included that have defined the use of mHealth to improve young people SRH services. mHealth is defined as medical and public health practice supported by mobile devices, such as mobile phones, patient monitoring devices, personal digital assistants (PDAs), and other wireless devices [19]. Studies assessing behavioral, health, and education, and awareness related outcomes through a range of mobile-based health interventions were included in the review. Additionally, studies were included that have identified common barriers and facilitators for the implementation of mHealth interventions for young people SRH. For young people SRH outcomes, the review utilized the United Nations Population Fund (UNPF) explanation which states that “Providing access to comprehensive sexuality education; services to prevent, diagnose and treat sexually transmitted infections (STIs); and counseling on family planning”. The UNPF also advocates that young people should be empowered so that they know their rights—including the right to delay marriage and the right to refuse unwanted sexual advances.

#### Type of studies

Randomized controlled trials (RCTs), non-randomized studies, pre- and post-test designs, non-experiment observational (cross-sectional, case-series, case studies) and qualitative papers, mixed methods studies were included in this review. Commentaries, editorials, symposium proceedings, and systematic reviews were excluded in this review as these are non‐empirical publications.

##### Time period

Studies published between January 1, 2005 and March 31, 2020 were included as the field of mHealth is recent and has emerged over the last decade. English language articles were included only as the authors are proficient in this Language. The inclusion and exclusion criterion is illustrated in Table 2.

**Table 2:** Eligibility criteria

Attribute	Inclusion criteria	Exclusion criteria
Population	Various terms are used to categorize young people: “adolescents” refers to 10–19 years; “youth” refers to 15–24 years; and “young people” refers to 10–24 years Studies involving young people (adolescents and youth) aged 10–24 years to which m-Health interventions were delivered for improving their SRH outcomes	Studies involving groups of women, men, and girls under the age of 10 years and over the age of 24 years
Intervention	Studies included that has involved mHealth intervention to improve ASRH services	Studies involving other ICT interventions, ART compliance reminders, EmONC coverage, managerial and financial level interventions, physical mobile clinics, and teleconsultations
Comparison	The comparison is the usual standard of care, or in the case of a randomized control trial, the comparison is the control condition	Not applicable
Outcome	Improvement in adolescent sexual and reproductive health services Behavioral outcomes Improved education and awareness ASR Health outcomes	Studies with other outcomes such as demonstrating skilled birth attendants, emergency care, quality of life, immunization coverage, the cost-effectiveness of the intervention, child development, and others
Setting	Studies conducted in LMICs	Studies conducted elsewhere
Study Designs	Randomized and non-randomized controlled trials, pre- and post-test designs, non-experiment observational (cross-sectional, case-series, case studies) and qualitative papers	Commentaries, editorials, symposium proceedings, systematic reviews
Language	Studies available in the English Language as authors are proficient in this language	Studies which were not available in English translation
Time period	Studies published between January, 2005 to March, 2018 as the field of mHealth emerged over the last decade	Studies published before January 2005 and after March 2018

### Information sources and search strategy

An electronic systematic literature search was carried out to explore the role of mobile Health technology in improving young people SRH, in LMICs. Although, there are a large number of databases on this pertinent topic; however, we searched four electronic databases including PubMed, CINAHL Plus, Science Direct platform and Cochrane Central as they are generally considered large databases in Medicine and are easily accessible and available. These databases were explored using detailed search strategy. Additionally, grey literature (non-published, internal or non-reviewed papers, repositories) was also explored as it is an important source for mHealth evaluations carried out in LMICs. The reference list of included records were also appraised to identify relevant articles. Moreover, the reference lists of identified systematic reviews were also reviewed to see if references include pertinent studies that might be included for review. The databases were searched by two researchers independently (AF and NAA). The search terms were grouped under five major categories of interest; population (youth, adolescents, young people), intervention (mHealth), barriers and facilitators for implementation of mHealth interventions for SRH services, outcome (SRH), and settings (LMICs). Additionally, indexed keywords in the Medical Subject Headings (MeSH) were used in order to ensure uniform search terms. The search strategy was piloted to ensure sufficient specificity and sensitivity. The detailed search strategy is illustrated in Table 3.

**Table 3:** Search strategy

Population	(‘adolescen*’ [Mesh] OR ‘school*age*’ OR student* OR teen* OR youth* OR ‘young adult*’ OR ‘young people’ OR ‘younger people’ OR ‘young women’ OR ‘young men’ ‘teenager’ OR ‘middle schooler’ OR ‘high schooler’ OR ‘secondary school’OR ‘Young adult’ [Mesh]) AND
Intervention	(Mobile phone OR mhealth[All Fields]) OR telemedicine[MeSH Terms]) OR cellphone[MeSH Terms]) OR reminder system[MeSH Terms]) OR wireless technology[MeSH Terms])OR text messaging[MeSH Terms]) OR medical informatics[MeSH Terms]) OR pda[MeSH Terms]) OR smartphone[MeSH Terms]) OR tablet computer[MeSH Terms]) AND
Outcome	(Health outcomes OR behavioral outcomes OR Education and awareness OR ‘sexual health’ OR ‘reproductive health’ OR ‘sexual behavior’ OR ‘sex education’ OR condom* OR HIV OR HIV/AIDS OR PLHIV OR “acquired immunodeficiency syndrome” OR HPV OR ‘family planning’ OR abortion* OR abstinen* OR contracept* OR pregnan* OR sexual health rights OR ‘sexually transmitted infection’ OR ‘sexually transmitted infections’ OR STI OR STIs OR ‘sexually transmitted disease’ OR ‘sexually transmitted diseases’ OR ‘STD’ OR ‘STDs’ OR ‘sexual debut’ OR puberty OR ‘safe sex’) AND
Setting	(‘Developing country’ OR ‘South Asian countries’ OR ‘African countries’ OR ‘low and middle income Arab Countries’ OR ‘developing nation’ OR ‘least developed country’ OR ‘least developed nation’ OR ‘less developed nation’ OR ‘third world country’ OR ‘third world nation’ OR ‘under developed country’ OR ‘remote region’ OR ‘low and middle income country’ OR ‘under developed nation’ OR ‘low and middle income nation’ OR Angola OR Indonesia OR Philippines OR Armenia OR Jordan OR São Tomé and Principe OR Bangladesh OR Kenya OR Solomon Islands OR Bhutan OR Kiribati OR Sri Lanka OR Bolivia Kosovo OR Sudan OR Cabo Verde OR Kyrgyz Republic OR Swaziland OR Cambodia OR Lao PDR OR Syrian Arab Republic OR Cameroon OR Lesotho OR Tajikistan OR Congo, Rep. OR Mauritania OR Timor-Leste OR Côte d'Ivoire OR Micronesia, Fed. Sts. Tunisia OR Djibouti OR Moldova OR Ukraine OR Egypt, Arab Rep. OR Mongolia OR Uzbekistan OR El Salvador OR Morocco OR Vanuatu OR Georgia OR Myanmar OR Vietnam OR Ghana OR Nicaragua OR West Bank and Gaza OR Guatemala OR Nigeria OR Yemen, Rep. OR Honduras OR Pakistan OR Zambia OR India OR Papua New Guinea)
Filters	Publication date from January 1, 2005 to March 31, 2020; Humans; English

### Study selection

Citation management system (Endnote software) was used to manage the records exported from all the electronic databases [20]. In order to ensure the reliability of screening articles among the two reviewers (AF and NAA), a pre-defined screening form was developed and pilot testing was conducted as per the eligibility criteria. Both reviewers (AF and NAA), described outcome measures after reviewing the studies to verify the relevance of the articles. Strong justifications for excluding studies were provided by each reviewer. Any disagreement between the two reviewers were resolved by a third reviewer (AK) in a consensus meeting. The third reviewer was consulted to make the final decision about whether the study meets the eligibility criteria for inclusion.

**Fig. 1:**
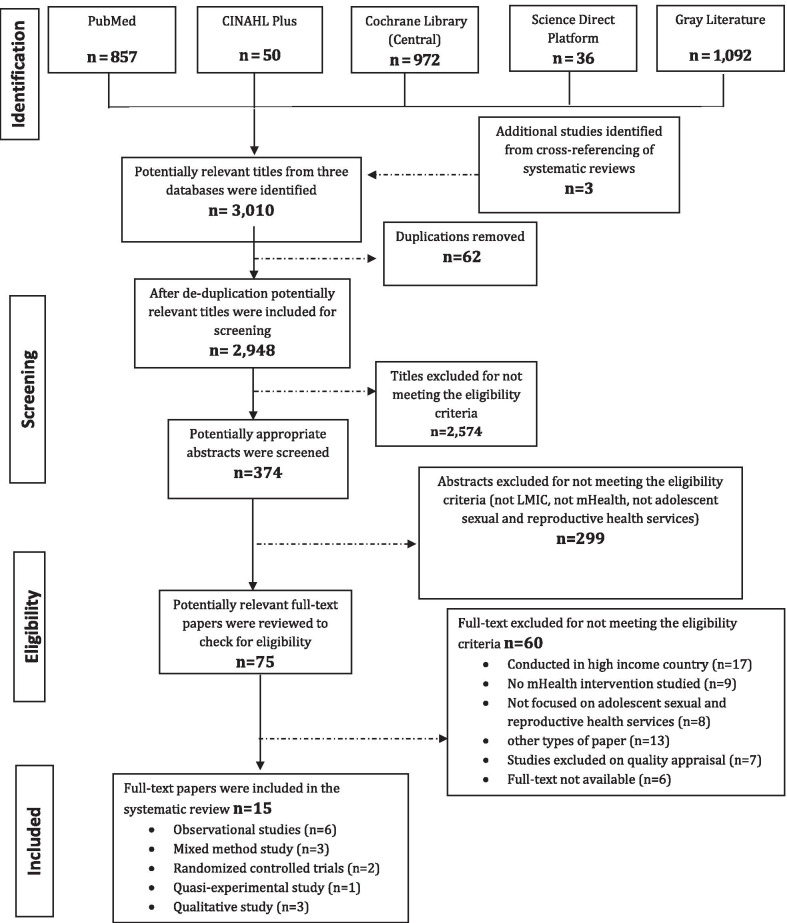
PRISMA flow diagram for database search of studies

All studies were first screened by titles, then by abstract, and finally by full text to progressively eliminate studies not meeting the inclusion criteria. Database searches identified a total of 3010 studies initially. After de-duplication, 2948 potentially relevant titles were included for title screening. After title screening, 374 records were screened by abstracts. Full texts of remaining 75 studies were reviewed to determine if they fulfill the inclusion criteria. Finally, 15 studies were selected and used for the purpose of this review [21–35]. The Preferred Reporting Items for Systematic Reviews and Meta-analyses (PRISMA) flow diagram was used to report the study selection process (Fig. 1).

### Quality assessment of included studies

To assess the methodological quality of the included studies, Mixed Methods Appraisal Tool (MMAT) was utilized. The tool was suited for this review as it was specifically developed for quality appraisal in systematic reviews involving qualitative, quantitative and mixed-methods designs. Qualitative and quantitative sections have four criteria each, and studies are scored by dividing the number of criteria met by four to arrive at a value ranging from 25 to 100%. For mixed method studies, we adapted the MMAT by assessing each segment separately and then selecting the lowest quality rating. Articles were not excluded based on MMAT score; the purpose was to examine and gain insight into the rigor of existing research in this field. Two reviewers (AF, NAA) independently assessed the quality of the included studies. Disagreements between reviewers were resolved by consensus or by the decision of a third independent reviewer (AK).. Data on quality appraisal is provided in an Additional File 1 for all the included studies.

### Data collection process

A customized data extraction sheet was filled by the two independent reviewers (AF, NAA) for all the included studies. Data extraction tables of both reviewers were matched to ensure that all key findings are included in the systematic review. Third evaluator (AK) was involved, if discordant information was observed during the data extraction process. The summary of included studies on mHealth interventions to improve young people SRH is provided in the Additional File 2.

The systematic review has been designed and reported according to the PRISMA checklist [36]. The systematic review protocol has been published [37] and registered in the ‘International Prospective Register for Systematic Reviews’ (PROSPERO) CRD42018087585 [38].

## Results

**Fig. 2:**
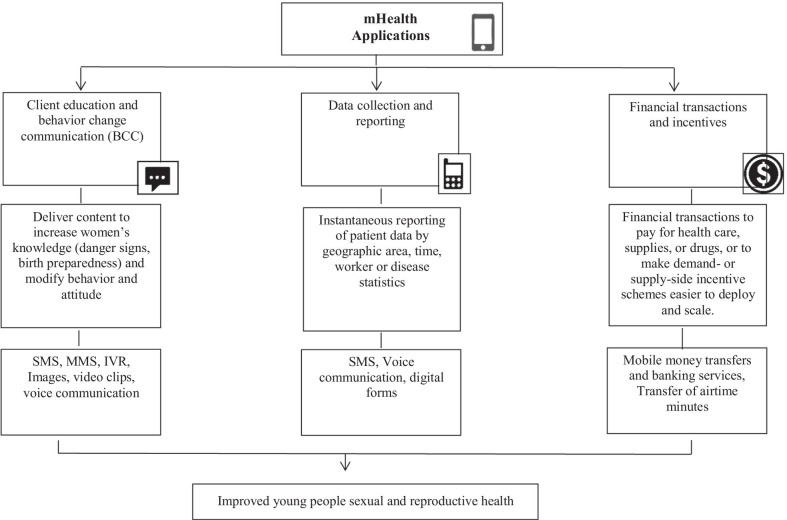
Conceptual Framework on mHealth Applications for young people sexual and reproductive health

The data from the final 15 studies fit in to the three key mHealth applications described in the Labrique and colleagues’ framework including, *client education and behavior change communication*, *data collection and reporting*, and *financial transaction and incentives*. All of these mHealth applications have been functioning using numerous mobile phone apps including “short message service (SMS), voice communication, and transfer of airtime minutes, e-credit for mobile account” [17]. The conceptual framework was adapted to elaborate the potential of mobile phones for improving young people SRH. The adapted framework is illustrated in Fig. 2.

### Type of studies

Out of fifteen studies which were included, six were observational studies [21, 23, 26, 29, 30, 32], two were RCTs [29, 34], three were mixed-methods study [24, 26, 27], one was quasi-experimental study and remaining three were qualitative studies [22, 31, 35]. All these studies included in the review were published within the time period from 2009 to 2020.

### Range of mHealth solutions

**Fig. 3:**
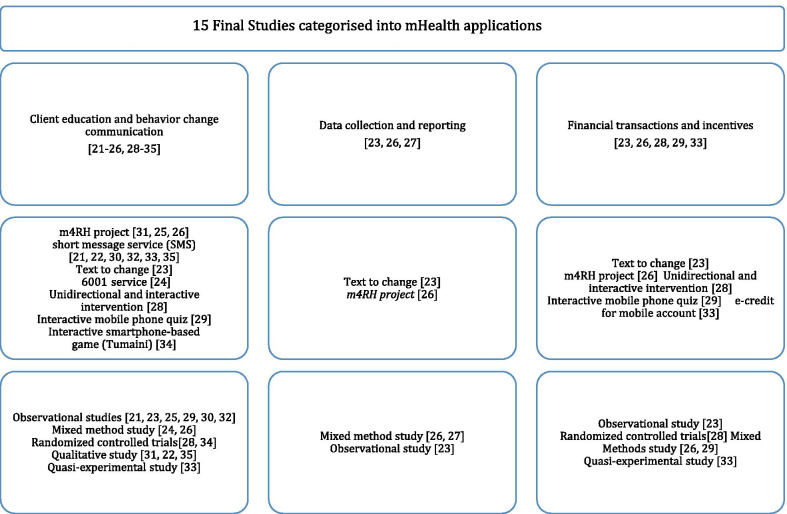
Classification of the Included Studies Based on the Types of mHealth Interventions Used

The 15 final studies were categorized according to the type of mHealth applications. While some studies addressed one mobile health application, many addressed multiple applications. Most studies were allocated in more than one mHealth application group if the intervention was multifarious. The final studies were generally characterized in three main applications which include client education and behavior change communication [21–26, 28–35], data collection and reporting [23, 26, 27], and financial transactions and incentives [23, 26, 28, 29, 33]. The results of the grouping exercise are illustrated in Fig. 3.

#### Client education and behavior change communication

Fourteen studies included in this review had ‘education and behavior change communication’ as one of the primary mHealth functions to improve young people SRH [21–26, 28–35]. Several studies highlighted that mobile phones are an effective tool to deliver HIV prevention educational program [21, 34], improve retention to HIV care and ART adherence for young people [35], maximize reach and access to family planning (FP) information [25, 31], improve young people reproductive health knowledge [28, 29, 33], sexual health knowledge and ensure safer sexual behavior [24, 32].

Three studies reported use of mobile phone interventions such as m4RH, text-based system, for improving access to family planning information. A qualitative study conducted at Dar es Salaam, Tanzania Nairobi, and Kenya obtained feedback on the feasibility of the m4RH project. The m4RH project is theorized as an automated, text-based system that is compatible with all mobile phones to improve access to family planning (FP) information via mobile phone. This study concluded that providing FP information via text message is a favorable method of reaching women and men with health information [31]. Another observational study was conducted to evaluate the feasibility of providing automated FP information via mobile phones m4RH to the general public in Tanzania. The study found out that 2870 unique users accessed m4RH in Tanzania, resulting in 4813 questions about contraceptive methods. A variety of changes in FP use were stated after using m4RH, with reported changes consistent with where the users are in their respective reproductive cycle. In Kenya, young people’s use of m4RH was examined through a mixed methods study. The study revealed that condom and natural FP information was retrieved most frequently, although users queried all FP methods. Overall, participants mentioned improved contraceptive knowledge and use after using m4RH [26]. Three studies examined the usage of mobile phones among adolescents to seek SRH health information and services. A qualitative study conducted in six Nigerian states, studied adolescent girls and young women’s reach and use of mobile phones to seek SRH information and services. The study concluded that there is high mobile phone access yet limited use of phones to access SRH information and services [22]. In India, a cross-sectional survey was conducted to study the level and pattern of mobile phone usage among adolescent girls. The study informed that most adolescent girls spent 2–4 h a day on an average using smartphones and 69% adolescents preferred SMS for awareness about reproductive and sexual health information [30]. In Ghana, a cross-sectional analytical study was conducted to measure use of mobile phone among adolescents and young adult populations and their use of these technologies in the education and prevention of STIs. The study found that of the 250 adolescents and young adults, 99% owned mobile phones and 58% of these were smartphones users. It was found that male young adults (Coef. = 1.11, p = 0.000) and young adults who owned a smartphone (Coef. = 0.46, p = 0.013) were more likely to use mobile phones for education and prevention of STIs [32].

Four studies examined the effectiveness of mHealth programs to improve SRH knowledge and ensure safe sexual behaviors among adolescents. A cluster RCT conducted in Ghana assessed whether text-messaging intervention can improve reproductive health among adolescent girls. A total of 38 schools were randomized to unidirectional intervention (n = 12), interactive intervention (n = 12), and control (n = 14). The unidirectional program sent SMS messages with reproductive health information. The interactive program involved teenagers in text-messaging reproductive health quiz games. The results showed large improvements in knowledge level at 3 months that were persistent after 15 months for both unidirectional and interactive interventions [28]. Another observational study conducted in Ghana assessed the degree to which mHealth interventions reach adolescent populations who may be at greater risk of poor SRH outcomes. The mHealth program included an interactive mobile phone quiz. The study concluded that mHealth programs are not only an effective tool in increasing SRH knowledge, but that these programs can also engage key target populations who are at greater risk of poor SRH outcomes, including adolescents with low parental education, adolescents with low SRH knowledge, adolescents with early sexual debut, and adolescents with low parental support [29]. A mixed-methods study conducted in Uganda implemented a mHealth intervention, to deliver reliable sexual health information, with the aim to improve sexual health knowledge and promote safer sexual behavior [24]. In Indonesia, a quasi-experimental study was conducted to evaluate the feasibility and acceptability of a text message intervention to improve young people’s knowledge of SRH. A total of 555 eligible young people were enrolled into the SMS intervention. The study concluded that the SMS intervention was feasible, acceptable and improved adolescents’ SRH knowledge between baseline and follow-up survey [2.7, (95% CI 2.47, 2.94) vs 3.4 (95% CI 2.99, 3.81) (p ≤ 0.01)][33].

Four studies assessed the usage of cell phones and effectiveness mHealth campaign for improving HIV/AIDS knowledge, prevention and treatment efforts. A cross-sectional study conducted in Uganda assessed cell phone use among 1738 adolescents aged 12 to 18 years, in an effort to understand if cell phones might have the potential for integration into HIV/AIDS prevention efforts. The survey found out that 27% adolescents have cell phones and about half (51%) of all students and 61% of those who owned a cell phone believe that they would access a text messaging-based HIV prevention educational program if it was available [21]. Another pilot effectiveness study conducted in Northwest Uganda, explored the efficacy of a mHealth campaign using SMS as a platform to disseminate and measure HIV/AIDS knowledge. The Text to Change HIV/AIDS education campaign was designed to increase knowledge about HIV/AIDS, awareness about the regional clinics and testing centers, and HIV testing behaviors in the Arua district of Uganda. The study concluded that the campaign had proportionately limited success in increasing knowledge on a mass scale because correct knowledge was only provided to respondents who answered questions (and people who answered incorrectly tended to answer fewer questions) [23]. In western Kenya, a RCT was conducted to assess acceptability of adolescent participants towards Tumaini intervention. Tumaini is a narrative-based smartphone game designed to help prevent HIV among young Africans aged 11 to 14 years by delaying first sex and increasing condom use at first sex. The study found strong acceptability of an interactive smartphone-based game to the adolescents. Also, the study reported that the adolescent participants were eager for additional content [34]. In Zambia, a qualitative study was conducted to explore barriers to HIV care and the acceptability and feasibility of using mHealth to improve retention into care and ART adherence for young people living with HIV (16–24 years old). The study found that twenty-four young persons had access to mobile phones and reported using them for social networking, information gathering and regular communication. The study concluded that participants are willing to use mHealth for improving retention into care and ART adherence in young people living with HIV[35].

#### Data collection and reporting

Three studies included in this review had data collection as one of the primary mHealth functions [23, 26, 27]. In Northwest Uganda, a pilot effectiveness study used ‘Text to Change’ campaign to achieve multiple objectives for public health. One of the main objectives of the campaign was to collect data on effectiveness of SMS-based campaigns in improving health care outcomes, specifically HIV knowledge. Thirteen questions were sent via SMS to collect data on three knowledge areas including (a) HIV/AIDS disease, (b) testing, and (c) HIV Counseling and Testing (HCT) services [23].

In Kenya, a mixed methods study was conducted to investigate young people’s use of m4RH, a text message-based contraception information service. The study employed three data collection methods to evaluate the acceptability, information access, and potential impact of providing contraception information via SMS to young people in Kenya. These include recording automatic logging of all m4RH system queries, demographic and behavior change questions sent via SMS to all users who accessed m4RH during the pilot period; and in-depth telephone interviews with a subset of m4RH users [26]. In Democratic Republic of Congo (DRC), a mixed methods study was conducted to understand the needs, expectations, and practices of teenagers in DRC urban areas concerning their sex and emotional life. Data was collected through an interactive radio show program *please doctor* in which old adolescents and young people participated by means of their cellphones. The study found that girls’ usually inquired information on menstrual cycle calculation, sexual practices, love relationships, and virginity. While boys’ asked questions related to masturbation, sexual practices, love relationships, and infections (genital and STI) [27].

#### Financial transactions and incentives

Five studies included in this review had used mHealth for financial transaction and incentive purposes [23, 26, 28, 29, 33]. In Northwest Uganda, the Text to Change HIV/AIDS education campaign was designed to increase knowledge about HIV/AIDS, awareness about regional clinics and testing centers, and HIV testing behaviors in the Arua district of Uganda. Between January 29 and February 27 2009, text messages with HIV/AIDS multiple choice and true/false questions were sent to 10,000 identified mobile phone numbers. Those participants who correctly answered questions received free HIV Counseling and Testing (HCT) services and were entered into weekly drawings to win prizes including mobile phones and airtime [23]. In Kenya, a mixed methods study was conducted to investigate young people’s use of m4RH, a text message-based contraception information service. In-depth telephone interviews with a subset of m4RH users to evaluate the acceptability, and potential impact of providing contraception information via SMS. Interviews lasted an average of 30 min, and participants received air time as an incentive for participation [26]. In Ghana, a cluster RCT was conducted to evaluate whether text-messaging programs can improve reproductive health among adolescent girls. The interactive intervention group received 1 multiple-choice quiz question via text message each week to which they were invited to respond free of charge. Upon responding, participants immediately received a confirmatory text message informing them whether they answered correctly along with the correct answer and additional information. For every 2 correct responses, participants were sent an airtime credit reward of 1 GHS (US$0.38) [28]. In Ghana, an observational study was conducted to assess the degree to which mHealth programs reach target adolescent subpopulations who may be at higher risk of poor SRH outcomes. The mHealth intervention included an interactive mobile phone quiz in which participants could win airtime (i.e. mobile phone credit that can be used for making calls or sending texts) for texting correct answers to SRH questions [29]. A quasi-experimental study was conducted in Indonesia to evaluate the feasibility and acceptability of a text message intervention to improve young people’s knowledge of SRH. A custom-built SMS gateway system was built for the purpose of this study, which managed all the SMS sending and any bouncing of messages. Enrolled participants received an initial welcome message with links to the baseline survey, followed by a series of 12 intervention messages, two per week delivered at the same time of the day. Following the intervention, participants were also invited to complete a follow-up survey (online only) and participants were given USD $2.50 worth of e-credit for their mobile account for completing each evaluation survey [33].

### Type of outcomes examined

#### Access to sexual and reproductive health services

Nine studies included in this review evaluated the impact of mobile health interventions to improve access to SRH services [21–23, 27, 29–33]. Most of these studies were largely conducted in African and Asian countries and used qualitative, quasi-experimental and observational study designs to understand the effect of mHealth technology on SRH education and services [21–23, 27, 29, 31–33]. Most studies examined the use of text messaging program to improve SRH services, while one study, used cell phone-based interactive radio show program to understand needs, expectations, and practices of teenagers concerning their sex and emotional life. Most studies reported positive outcomes such as, improved access to family planning information due to automated text-based system, improved sexual and reproductive health knowledge among adolescents through text-messaging program, willingness to use SMS for awareness about reproductive and sexual health information, and readiness to access a text messaging-based HIV prevention educational program [21, 27, 29–33]. However, only two studies reported unfavorable outcomes such as, restricted use of phones to access SRH information and services and limited success to increase SRH knowledge levels on a mass scale via mHealth campaign [22, 23].

#### Sexual and reproductive health outcomes

Six studies examined the impact of mHealth solutions on SRH outcomes [24–26, 28, 34, 35]. Studies largely used mixed methods, RCTs, qualitative study and observational study designs to assess the feasibility of mHealth programs to improve SRH outcomes. The studies reported use of different type of mHealth programs to improve SRH outcomes such as, m4RH, interactive mobile phone quiz, smartphone game and health information mobile intervention [24–26, 28, 34]. Most studies reported positive outcomes such as, improved family planning knowledge and use, prevention of HIV among young Africans, improved retention into HIV care and ART adherence and increased involvement of target populations, who are at higher risk of poor SRH outcomes, through interactive mobile phone quiz [25, 26, 28, 34, 35]. Unexpectedly, one study reported limited success in increasing SRH knowledge, and changing attitudes including risky sexual behaviors, and infidelity, as a result of mHealth intervention [24].

#### Factors facilitating and impeding uptake of mHealth interventions for young people sexual and reproductive health

Out of 15 final studies, three studies reported benefits of using mHealth services for improving SRH. In Kenya, young people’s use of m4RH was investigated through a mixed methods study. Study participants perceived m4RH as confidential, convenient, and a valuable source of contraception information outside of the clinic setting [26]. Another study conducted separately reported the benefits of SMS and voice call, perceived by the study participants. The major factors facilitating the use of mHealth solutions include confidentiality, secrecy, quick and easy correspondence, easy retrieval of information, etc. [22]. A qualitative study conducted at Dar es Salaam, Tanzania Nairobi, and Kenya obtained feedback on the feasibility, design, and content of the m4RH project. The participants appreciated the m4RH service and preferred to use it in the future as it ensures privacy and address stigma related issues [31].

Only two studies reported barriers to uptake of mHealth interventions for SRH. A study conducted in Ghana reported barriers pertinent to mHealth such as decreased technological literacy, inferior network coverage, and lower linguistic competency [29]. Another study conducted in six Nigerian states, examined adolescent girls and young women’s access and use of mobile phones, to seek SRH information and services. The study reported several barriers to mHealth services utilization including cost of service, request for socio-demographic information that could break anonymity, poor marketing and publicity, socio-cultural beliefs and expectations of young girls, individual personality and beliefs, as well as infrastructural/network quality [22].

## Discussion

The review reports evidence on the range of mHealth applications used at different levels of the healthcare system for improving young people SRH in urban and rural communities of LMICs. Most of the studies took place in East Africa and West Africa, while few were undertaken in Central Africa, Sub Saharan Africa, South Africa and South Asia. The mHealth solutions identified in this systematic review mainly aimed to improve contraception related SRH education, services and outcomes, for young people. A Cochrane review on mobile phone-based interventions for improving contraceptive use also suggests that a series of voice messages and daily educational text messages can improve continued use of contraceptive pill among young adults [39]. In addition, this review found that text message-based health interventions are very feasible, and acceptable for improving SRH knowledge among Indonesian adolescents. Notably, the current evidence base shows some promise for the use of similar SMS-based interventions for improving young people SRH knowledge and services in other LMICs. Labrique and colleagues’ framework was adapted for categorizing the mHealth interventions according to their purpose. Based on our analysis, the most reported use of mHealth was for client education and behavior change communication [21–26, 28–35], followed by data collection and reporting [23, 26, 27], and financial transactions and incentives [23, 26, 28, 29, 33]. The categorization of the studies in to various mHealth applications provided the understanding that the strongest evidence exists on client education and behavior change communication mHealth application. These findings are in concordance with the other reviews, which suggests, that mobile phone approaches; including texting in particular, have been explored much by various studies as it provides feasible and potential efficacious medium for increasing levels of reproductive and sexual health education [40]. Little evidence exists on other type of mHealth applications such as, sensors and point-of-care diagnostics, registries/ vital events tracking, electronic decision support, and supply chain management. Thus, a more complete understanding of the role of mobile phones for improving young people SRH is required, to strengthen the evidence base in overlooked areas.

As with most reviews in the emerging field of mHealth, this review is limited by the difficulty of analyzing complex intervention studies and the variety of different interventions across included studies. More studies are needed to refine the current work with a larger body of evidence and to establish how best to integrate it with the published existing framework. The heterogeneity of the interventions and outcomes measures restricted the interpretation through meta–analyses. The studies did not utilize a related taxonomy for explaining the range of mHealth application. In addition, several studies combined multiple mHealth interventions [23, 26, 28, 29, 33], making it challenging to determine to what degree each intervention contributed towards the expected outcome.

Overall, most studies included in this review were of moderate quality, indicating the significance of increasing the methodological rigor of future research.

The review has provided an understanding of how mHealth solutions targeted to youth population help address issues of ‘provider prejudice, stigmatization, discrimination, fear of refusal, lack of privacy and confidentiality, cost prohibitions, and transportation challenges’ [22, 26, 31]. Simultaneously, the review has highlighted the barriers to uptake mHealth solutions for SRH including poor technological literacy, inferior network coverage, and lower linguistic competency, high cost of service, and socio-cultural beliefs and expectations which does not favor the use of mHealth [22, 29]. Similar to other reviews, this paper recommends that more understanding is needed about the challenges of data privacy, technological literacy, linguistic competency and phone access to address the barriers impeding the uptake of mHealth for improving young people SRH information and services [16]. It is also important to note that each LMIC will face different challenges relating to implementation of unique mHealth interventions and thus the adoption strategies may vary among different LMICs. This opens a window of opportunity to look at the issue in a broader perspective with the intension to explore most important challenges of technology implementation.

## Conclusion

The review provides insights for the research community and public health professionals in making decisions regarding the use of innovative, engaging and effective mobile phone interventions to improve young people SRH outcomes, yet the room remains for additional evidence and innovation in overlooked areas. Finally, as the field of mHealth is maturing, additional research would be beneficial to discover the cost-effectiveness of mHealth interventions for improving SRH services and outcomes for young people.

## Supplementary Information

**Additional File 1:** Data on quality appraisal is provided in an additional file 1 for all theincluded studies

**Additional File 2:** The summary of included studies on mHealth interventions to improve young people SRH

## Data Availability

Not applicable.

## Abbreviations

STI: Sexually transmitted infections
GNI: Gross National Income
SRH: Sexual and Reproductive Health
LMICs: Lower-Middle-Income Countries
HIC: High Income Countries
PRISMA: Preferred Reporting Items for Systematic Reviews and Meta-analyses
RCTs: Randomized Controlled Trials
WB: World Bank
UNPF: United Nations Population Fund
