# Supplementary material for: Using mobile phones to improve young people sexual and reproductive health in low and middle-income countries: a systematic review to identify barriers, facilitators, and range of mHealth solutions
Source: Reprod Health. 2021 Jan 16;18:9. doi: 10.1186/s12978-020-01059-7 (PMC7811742; doi:10.1186/s12978-020-01059-7)
Supplement: Supplementary file 1 — Additional File 1: Data on quality appraisal is provided in an additional file 1 for all theincluded studies [file 12978_2020_1059_MOESM1_ESM.docx]

**Result of quality appraisal, the Mixed Methods Appraisal Tool (MMAT)**

1. **Qualitative study**

| Qualitative studies | Quality assessment components using MMAT | | | | |
| --- | --- | --- | --- | --- | --- |
|  | Are the sources of data relevant to research question? | Is the data analysing process relevant to address research question? | Is appropriate consideration given to how findings relate to context? | Is appropriate consideration given to how findings relate to researchers’ influence? (Reflexivity) | Total |
| Akinfaderin-Agarau, F., et al., 2012 | * | * | * | * | 4* |
| L'Engle et al, 2009 | * | * | * | * | 3* |
| [Natalie](https://www.ncbi.nlm.nih.gov/pubmed/?term=St%20Clair-Sullivan%20N%5BAuthor%5D&cauthor=true&cauthor_uid=31620472)  CS, et al 2019 | * | * | * | * | 4* |
| Total=4 |  |  |  |  |  |

1. **Quantitative non-randomized studies**

| Quantitative non-randomized studies | Quality assessment components using MMAT | | | | |
| --- | --- | --- | --- | --- | --- |
|  | Are participants recruited in a way that minimizes selection bias? | Are measurements appropriate  regarding the exposure or intervention and outcomes? | In the groups being compared, are the participants  comparable, or do researchers take into account? | Are there complete outcome data (80% or above), an acceptable response rate (60% or above), or an acceptable  follow-up rate for cohort studies? | Total |
| Singh & Jain, 2017. | * | * | * | * | 4* |
| Slawa & Fink , 2017 | * | * | * | * | 4* |
| Engle, K.L., et al, 2013 | 0 | * | * | * | 3* |
| Chib, A., et al 2012 | 0 | * | * | * | 3* |
| Mitchell, K.J., et al, 2012 | * | * | * | * | 4* |
| Nsakala, G.V., et al, 2014 | * | * | * | * | 4* |
| Robert K A., et al, 2019 | * | * | * | * | 4* |
| Alisa Pedrana., et al, 2020 | * | * | * | * | 4* |
| Total = 8 |  |  |  |  |  |

1. **Mixed Methods studies**

| Qualitative studies | Quality assessment components using MMAT | | | | |
| --- | --- | --- | --- | --- | --- |
|  | Are the sources of data relevant to research question? | Is the data analysing process relevant to address research question? | Is appropriate consideration given to how findings relate to context? | Is appropriate consideration given to how findings relate to researchers’ influence? (Reflexivity) | Total |
| Vahdat, H.L., et al., 2013 | * | * | * | * | 4* |
| Jamison et al., 2013 | * | * | * | * | 4* |
| Total = 2 |  |  |  |  |  |

| Qualitative descriptive component | Quality assessment components using MMAT | | | | |
| --- | --- | --- | --- | --- | --- |
|  | Is the sampling strategy relevant to address the quantitative research question? | Is the sample representative of the population under study? | Are measurements appropriate (clear origin, or validity known, or standard instrument)? | Is there an acceptable response rate (60% or above)? | Total |
| Vahdat, H.L., et al., 2013 | * | * | * | * | 4* |
| Jamison et al., 2013 | * | * | * | * | 4* |
| Total = 2 |  |  |  |  |  |

| Mixed method design | Quality assessment components using MMAT | | |  |
| --- | --- | --- | --- | --- |
|  | Is the mixed methods research design relevant to address the qualitative and quantitative research  questions (or objectives), or the qualitative and quantitative aspects of the mixed methods question (or  objective)? | Is the integration of qualitative and quantitative data (or results) relevant to address the research  question (objective)? | Is appropriate consideration given to the limitations associated with this integration, e.g., the  Divergence of qualitative and quantitative data (or results)? | Total |
| Vahdat, H.L., et al., 2013 | * | * | 0 | 2* |
| Jamison et al., 2013 | * | * | 0 | 2* |
| Total = 2 |  |  |  |  |

1. Randomized control studies

| Randomized control studies | Quality assessment components using MMAT | | | | |  |
| --- | --- | --- | --- | --- | --- | --- |
|  | Is randomization appropriately performed? | Are the groups comparable at baseline? | Are there complete outcome data? | Are outcome assessors blinded to the intervention provided? | Did the participants adhere to the assigned intervention? | Total |
| Rokicki, S., et al., 2017 | * | * | * | 0 | * | 4 |
| Gaëlle, S., et al, 2019 | 0 | * | * | 0 | * | 3 |
| Total= 2 |  |  |  |  |  |  |
